# Controlled Release of Metformin Hydrochloride from Core-Shell Nanofibers with Fish Sarcoplasmic Protein

**DOI:** 10.3390/medicina55100682

**Published:** 2019-10-10

**Authors:** Su Sena, Korkmaz Nalan Sumeyra, Guven Ulkugul, Arslan Sema, Karademir Betul, Sennaroglu Bostan Muge, Eroglu Mehmet Sayip, Uzun Muhammet, Kalkandelen Cevriye, Mahirogullari Mahir, Aurel Mihail Titu, Anton Ficai, Denisa Ficai, Oguzhan Gunduz

**Affiliations:** 1 Center for Nanotechnology & Biomaterials Application and Research, Marmara University, 34722 Istanbul, Turkey; 2 Department of Metallurgical and Materials Engineering, Faculty of Technology, Marmara University, 34722 Istanbul, Turkey; 3 Department of Genetics and Bioengineering, Faculty of Engineering, Yeditepe University, 34755 Istanbul, Turkey; 4 Department of Biochemistry, Marmara University, 34854 Istanbul, Turkey; 5 Department of Chemical Engineering, Faculty of Engineering, Marmara University, 34722 Istanbul, Turkey; 6 Department of Textile Engineering, Faculty of Technology, Marmara University 34722 Istanbul, Turkey; 7 Department of Biomedical Devices Technology, Vocational School of Technical Sciences, Istanbul University—Cerrahpasa, 34500 Istanbul, Turkey; 8 Nanortopedi Industry and Trade Inc., Sanayi mahallesi Teknopark Bulvari, Teknopark Istanbul, 34906 Istanbul, Turkey; 9 Industrial Engineering and Management Departament, “Lucian Blaga” University of Sibiu, Faculty of Engineering, 4 Emil Cioran Street, 550025 Sibiu, Romania; 10 Academy of Romanian Scientists, 54 Splaiul Independentei, Sector 5, 50085 Bucharest, Romania; 11 Department of Science and Engineering of Oxidic Materials and Nanomaterials, Faculty of Applied Chemistry and Materials Science, University Politehnica of Bucharest, 011061 Bucharest, Romania

**Keywords:** coaxial electrospinning, fish sarcoplasmic protein, nanofibers, wound healing

## Abstract

*Background and Objectives*: A coaxial electrospinning technique was used to produce core/shell nanofibers of a polylactic acid (PLA) as a shell and a polyvinyl alcohol (PVA) containing metformin hydrochloride (MH) as a core. *Materials and Methods*: Fish sarcoplasmic protein (FSP) was extracted from fresh bonito and incorporated into nanofiber at various concentrations to investigate the influence on properties of the coaxial nanofibers. The morphology, chemical structure and thermal properties of the nanofibers were studied. *Results*: The results show that uniform and bead-free structured nanofibers with diameters ranging from 621 nm to 681 nm were obtained. A differential scanning calorimetry (DSC) analysis shows that FSP had a reducing effect on the crystallinity of the nanofibers. Furthermore, the drug release profile of electrospun fibers was analyzed using the spectrophotometric method. *Conclusions*: The nanofibers showed prolonged and sustained release and the first order kinetic seems to be more suitable to describe the release. MTT assay suggests that the produced drug and protein loaded coaxial nanofibers are non-toxic and enhance cell attachment. Thus, these results demonstrate that the produced nanofibers had the potential to be used for diabetic wound healing applications.

## 1. Introduction

Wound healing is a complex, dynamic, continuous, and multicellular process which targets barrier repair in the skin. This process involves many factors, such as extracellular matrix (ECM), blood cells, growth factors, and cytokines. Thus, it is difficult to obtain excellent results in regeneration of the tissue due to the these factors and pathological and physiological complexity of this process [1,2].

Nanofibers can provide a favorable environment for wound healing due to their similarity to the extracellular matrix diameters, high porosity, high gas permeability, and high surface area-volume ratio. These advantages support skin regeneration, cell respiration, moisture retention, and hemostasis in the wound healing process [3,4]. The electrospinning method is a suitable method for the production of nanofibers because of the simplicity, the ability to produce nanofibers from different combined substances, the highly applicability and the ability of the scaffold’s properties to be adjusted by changing the parameters of the method [5,6]. In general, the basic principle of the electrospinning method is to create a taylor cone by applying an electrical field to the polymer solution at the tip of the needle. When the force applied to the surface of the liquid is stronger than the surface tension, the polymer solution moves toward the grounded target electrode. Thus, the polymers are accumulated in the collecting area for the nanofiber formation [7].

The coaxial technique is the electrospinning of simultaneous and coaxial capillaries to form the core-shell structure of two different components in nanofiber production [8]. The nanofibers produced by the coaxial technique have many advantages in the field of drug delivery such as the high encapsulation efficacy, the high diversity in the selection of drugs and materials, the simple procedure, cheapness and providing control over the drug release [1]. When compared to the blending technique, the core-shell structure of coaxial nanofibers prevents the burst of the drug and the drug release can be adjusted by changing the thickness of the shell [9,10].

Natural polymers (carbohydrates, proteins, and lipids) are widely used in nanomaterial production [11,12,13]. Proteins have more advantages than other natural polymers, such as their biocompatibility, which is important in many cell processes (cell signaling, cell adhesion, and the cell cycle etc.), their ability to catabolise biochemical reactions, and their ability to improve synthetic biomaterials by the structural and functional properties of fibrous proteins [14]. The fish sarcoplasmic proteins (FSP) can be obtained in the waste water from the fish industry which constitute 25–30% of the fish muscle proteins. FSP is a heterogeneous protein mixture which includes proteins and peptides with a molecular weight of up to 200 kDa [15].

The use of synthetic polymers in combination with natural polymers results in higher mechanical properties and more flexible nanofibers [16]. Polylactic acid (PLA) polymer is a biocompatible, biodegradable, flexible, durable polymer that has enhanced mechanical characteristics and it is an environmentally friendly composite. Due to these properties, PLA is suitable for wound dressing applications [17]. The carrier polymers are used in order to serve in carrying the bioactive substance (protein etc.) and the drugs [18]. Polyvinyl alcohol (PVA) is a water soluble polymer which has great physical and chemical properties, such as non-toxicity, processability, highly biocompatibility, a wide range of crystallinity, good film formation capacity, biodegrability and high crystal modulus [8].

The wounds caused by diabete disease are chronic, difficult to treat, and are most common in the feet [19]. Metformin Hydrocloride (MH) is the most widely used a biguanide drug in the treatment of type 2 diabetes and diabetic ulcers [20]. Metformin is suitable for wound treatment and local drug delivery after the encapsulation process [21].

Although many protein types, such as soy protein, whey, and keratin, have been used in drug release studies, there have been few empirical investigations into FSP protein in the use of drug delivery [22,23]. Therefore, this study makes a major contribution to research on the effects of FSP on drug delivery systems. The objective of this study was to develop a biodegradable core-shell nanofibers with polylactic acid as a shell. Polyvinyl alcohol containing MH and fish sarcoplasmic protein as a core were produced by a coaxial electrospinning technique. Nanofibers were prepared using various amounts of FSP to investigate the influence of the FSP on the morphological and chemical basis and to examine drug release properties of the coaxial nanofibers. The produced nanofibers were characterized by scanning electron microscopy (SEM), fourier-transform infrared spectroscopy (FTIR) and differential scanning calorimetry (DSC). The in vitro drug release profile of nanofibers was evaluated by UV-VIS spectroscopy and cell viability examined via MTT assay.

## 2. Materials and Methods

### 2.1. Sarcoplasmic Protein (SP) Preparation

Fish sarcoplasmic protein (FSP) was extracted from fresh bonito by using the method presented in our previous study [24]. The bonitos stored at −30 °C were first thawed and chopped. They were homogenized with deionized water and then centrifuged for 15 min, 18,000 × *g*, 5 °C. After the centrifugation, the supernatant was freeze dried (Alpha 1–2 LD Plus; Christ, Osterode am Harz, Germany) for obtain the FSP powder. This study was carried out under protocol number FEN-B-121218-0614 obtained on 15 June 2018.

### 2.2. Preparation of Spinning Solutions

PLA solution was prepared at a concentration of 8% (*w*/*v*) in DCM:DMF (4:1 *v*/*v*) and stirred for about 1 h to obtain viscous solution. PVA solutions at 5% (*w*/*v*) of polymer content were prepared in distilled water and stirred at 80 °C for 4 h to ensure complete dissolution. Then, the solution was cooled down to room temperature, 1 wt%, 3 wt% and 5 wt% FSP contents and 2% (*w*/*v*) metformin hyrochloride (MH) (20 mg) were individually added into the PVA solution under constant stirring. Contents of the core and the shell spinning solutions of the samples is given in Table 1.

### 2.3. Coaxial Electrospinning

The nanofibers were produced by using a laboratory scale electrospinning unit (NS24, Inovenso Co., Istanbul, Turkey). Two individual syringes containing the core (PVA solution) and the shell (PCL solution) were connected to a coaxial spinneret and driven by two separate pumps. A voltage of 13 kV was supplied by a high-voltage power supplier during coaxial electrospinning. The distance between the coaxial nozzle and the grounded collector was set as 12 cm, the flow rate of PLA solution was 1.4 mL/h, and the PVA solution was 0.5 mL/h. All of the electrospinning processes were carried out under ambient conditions (25 °C with a relative humidity of 55%).

### 2.4. Protein Composition of FSP

The protein composition of the freeze-dried fish sarcoplasmic protein (FD FSP) was analyzed by a sodium dodecyl sulfate polyacrylamide gel electrophoresis (SDS-PAGE) method using 5% (*w*/*v*) stacking gel and 12% (*w*/*v*) resolving gel according to the method of Laemmli (1970). An amount of 27.5 mg FD FSP sample was dissolved in 0.5 mL of demineralised water. It was then centrifuged and after centrifugation, the supernatant was used to determine the protein composition. The electrophoresis procedure was performed at 100 V for 15 min and at 120 V for 75 min by adjusting to pH 8.3 in tris glycine buffer. Samples were boiled for the denaturation procedure before the loading onto the gel. After the electrophoresis, proteins in the separating gel were determined by coomassie staining. Fermentas Protein Molecular Weight Marker (Pierce™ Unstained Protein MW Marker, Thermo Fisher Scientific, Waltham, MA, USA) containing seven proteins within 14.4–116 kDa range was used in order to determine the molecular weight range of the proteins by the Image Analyzer System (Chemi Doc MP Imaging System-BioRad, Hercules, CA, USA).

### 2.5. Scanning Electron Microscopy (SEM)

The morphologies of the electrospun fibers were examined using a scanning electron microscope(SEM) (EVA MA 10, Zeiss, San Diego, CA, USA) at an accelerating voltage of 10 kV. Samples were coated with gold/palladium (18 nm) using a sputter coater (SC7620, Quorum, Lewes, UK). The average fiber diameters and their distribution were determined by the image analysis software (SmartSEM, Zeiss, San Diego, CA, USA) using the measurement of 100 fibers selected randomly from each sample.

### 2.6. Fourier-Transform Infrared Spectroscopy 

To determine the composition and possible interaction between the fibers components, the infrared spectrums of the fibers were registered using a fourier-transform infrared spectroscopy (FT-IR) (FT/IR 4700, Jasco, Tokyo, Japan) equipped with a Gladi attenuated total reflection (ATR) viewing plate (Diamond ATR crystal) and a liquid-nitrogen cooled mercury cadmium telluride (MCT) detector at a wavelength of 500 cm^−1^ to 4000 cm^−1^. The nanofibers used in the drug release study for 21 days were dried at vacuum oven at 40 °C for one day before the FTIR test.

### 2.7. MTT Assay

The hacat cell lines, which is human keratinocyte cell line purchased from the American Type Culture Collection (ATCC, Manassas, VA, USA) were plated separately in tissue culture flasks and cultured in Dulbecco’s Modified Eagle Medium (DMEM) containing 10% fetal bovine serum (FBS). The cells were maintained in a humidified incubator at 37 °C with 5% CO_2_ and they were trypsinized and counted with a hemocytometer. All the nanofibers were cut to a size of 1 cm^2^ and sterilized in UV. DMEM cell culture medium containing 10% FBS, 1% Penicillin/Streptomycin as well as 100,000 cells were then seeded onto nanofibrous membranes in 24-well culture plate.

MTT (3-(4,5-dimethylthiazol-2-yl)-2,5-diphenyl tetrazolium bromide) was dissolved in PBS at 5 mg/ml. The stock MTT solution (66 µl per well) was added on the cells and materials in DMEM supplemented with 10% FBS, 1% Penicillin/Streptomycin in the culture plate incubated for 2 h at 37 °C and 5% CO_2_. After incubation, purple colored formazan crystal was formed at the bottom of the well and the above DMEM was withdrawn. DMSO solution was added to wells and measured for light absorbance in 96 wells/plate at 570 nm.

### 2.8. In Vitro Release Studies 

In vitro drug release was performed by the metformin hydrochloride which was loaded into the PVA/PLA electrospun nanofibers. The drug loaded nanofibers were cut into 2 × 2 cm pieces, weighted and immersed in glass bottles containing 7 mL of phosphate buffer solution (PBS) and the pH of the PBS solution was adjusted to 7.4 then shaked at 37 °C for 21 days. At determined time intervals, the amount of drug relase was measured at 233 nm by UV-VIS spectrophotometer. Each experiment was repeated three times.

### 2.9. In Vitro Release Kinetics

To disclose the drug release mechanism, the MH release profiles were fitted to four popular mathematical equations such as zero order, first order, Higuchi and Peppas-Korsemeyer equations represented as Equations (1)–(4).

Zero order kinetic defines a linear relationship between the fractions of drug release versus time:

*Q* = *K_0_t* (1)

where *Q* is the fractional amount of drug release at time *t* and “*K_0_*” is the zero-order release constant.

The equation that describes first order kinetic is

*In(1 − Q)* = −*K_1_t* (2)

where *Q* is the fractional amount of drug release at time *t* and *K_1_* is the first-order release constant.

Higuchi kinetic defines a linear dependence of the active fraction released per unit of square root of time:

*Q* = *K_h_t^1/2^* (3)

where *Q* is the fractional amount of drug release at time *t* and *K_h_* is the Higuchi kinetic constant.

The equation of Korsmeyer–Peppas is

*Q* = *Kt^n^* (4)

where *Q* is the fractional amount of drug release at time *t*, *K* is the kinetic constant and *n* is the diffusion exponent which is indicative of the drug release mechanism.

### 2.10. Thermal Analysis

The thermal properties of the nanofibers were analyzed using a Perkin Elmer Jade differential scanning calorimeter (DSC). Samples were weighed and placed in alumina pans. The heating was performed from room temperature to 300 °C with a heating rate of 10 °C/min under dynamic argon atmosphere (20 mL/min).

## 3. Results

### 3.1. SDS-PAGE Assay

**Figure 1:**
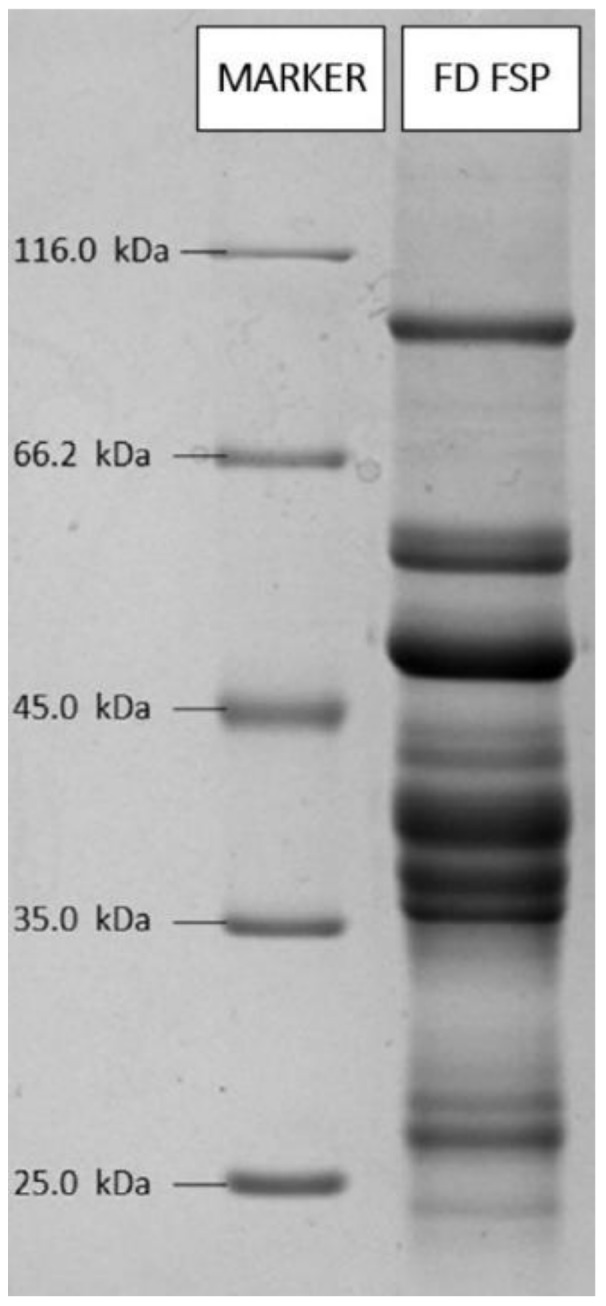
Protein pattern of freeze dryed fish sarcoplasmic protein.

SDS-PAGE (sodium dodecyl sulphate-polyacrylamide gel electrophoresis technique) is commonly used technique for characterization of proteins. Molecular weight distrubition of FSP were determined. As illustrated in Figure 1, with molecular weights (MW) ranging from 23 to 94 kDa were observed and it was identified that the 3840 and 48 kDa patterns were the most intense ones. These observations are mainly consistent with other studies [25,26]

### 3.2. Morphological Characterization of Nanofibers

**Figure 2:**
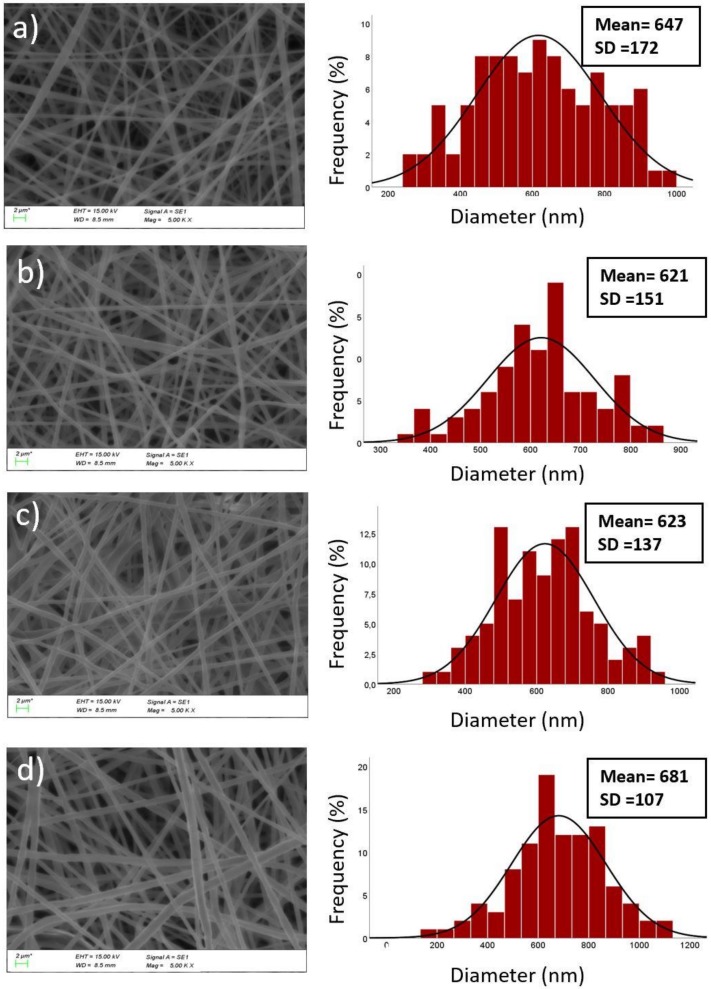
SEM images and fiber diameter distribution of electrospun nanofibers (**a**) MH (**b**) MHFSP1 (**c**) MHFSP3 (**d**) MHFSP5.

SEM images and associated diameter histograms of the prepared nanofibers having different concentrations of FSP are shown in Figure 2. The core/shell nanofibers possess a smooth surface, which indicates a successful encapsulation of FSP and MH without deteriorating the morphology of the fibers. It was observed from the SEM images that all samples are defect-free, beadless, and geometrically uniform nanofibers. However, fiber diameter and fiber distribution were changed by the addition of protein and increasing its concentration. As shown in Figure 2, average diameters of MH, MHFSP1, MHFSP3, MHFSP5 were recorded as 647 nm, 621 nm, 623 nm, and 681 nm, respectively. The average diameter were affected by voltage value. Therefore, the constant voltage applied during nanofiber formation to examine the only FSP content affected the diameters [27,28]. Clearly, the addition of FSP decreased the average fiber diameters and the standard deviation. This can be attributed to the higher electrical conductivity of FSP, which caused much more charge over the ejected jet and thus, higher elongation. Furthermore, decreasing the standart deviation by the amount of FSP was due to highest chain entanglements in the core. The observed increase in the diameters of the nanofibers with an increase in the FSP content from %1 to %5 can be associated with the knowledge that an increase in solution concentration usually results in a larger fiber diameter. These observations are consistent with those of previous studies performed with different globular proteins [15,16,17,18,19,20,21,22,23,24,25,26,27,28,29,30]

### 3.3. Fourier-Transform Infrared Spectroscopy (FTIR)

**Figure 3:**
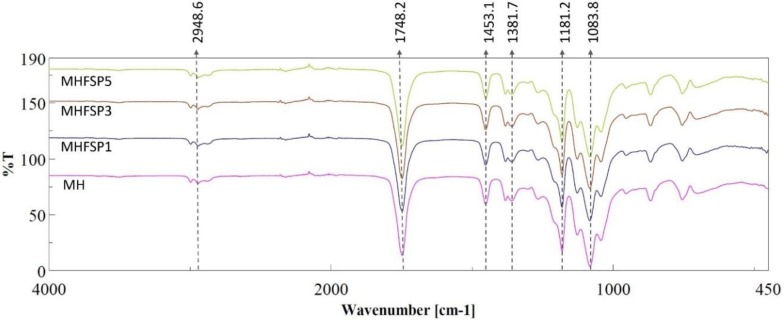
Fourier-transform infrared spectroscopy (FTIR) spectra of the MH, MHFSP1, MHFSP3 and MHFSP5 nanofiber samples.

**Figure 4:**
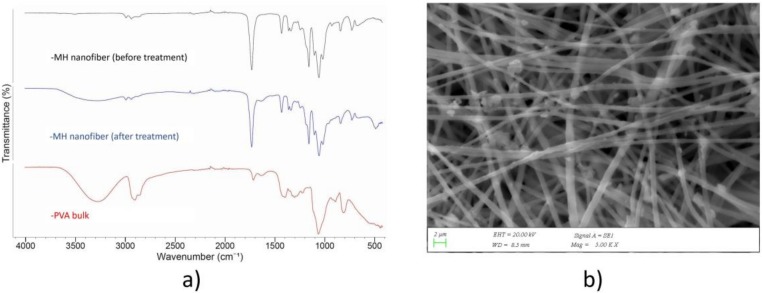
(**a**) FTIR spectra of PVA bulk, MH nanofiber before and after phosphate buffer solution (PBS) treatment; (**b**) Scanning electron microscopy (SEM) image of the MH nanofiber after the PBS treatment.

FTIR spectroscopic imaging in ATR (Attenuated Total Reflection) mode permits investigation of the molecular and structural properties of polymers [31]. FTIR spectra of MH, MHFSP1, MHFSP3, MHFSP5 core-shell fibers are depicted in Figure 3. The spectra of nanofibers show the general PLA bands, including the CH^3^ asymmetric stretching at 2948.6 cm^−1^, C=O stretching at 1748.2 cm^−1^ and C-0=0 stretching at 1181.2 cm^−1^ and 1038.8 cm^−1^, which is a characteristic ester bond. Bending vibrations for CH^3^ asymmetric and CH^3^ symmetric were identified at 1453.1 cm^−1^ and 1381.7 cm^−1^, respectively [32,33]. PVA shows the typical infrared bands of hydrocyl groups at 3200–3600 cm^−1^ which can not be seen in the core-shell structured nanofibers. This indicates that the PVA is not on the surface of the fibers, which is an additional proof of effective encapsulation in core-shell nanofibers [34]. After the PBS treatment, typical infrared bands of hydrocyl groups of PVA at 3200–3600 cm^−1^ were observed in the nanofibers and FTIR spectra of MH nanofiber are shown in Figure 4a as an example. The SEM image of MH nanofiber is also seen in Figure 4b, and a morphological difference is clearly observed before and after PBS treatment. These observations indicate that distruption of the PLA structure on the surface of the nanofibers provide the presence of PVA on the core.

### 3.4. MTT Assay

**Figure 5:**
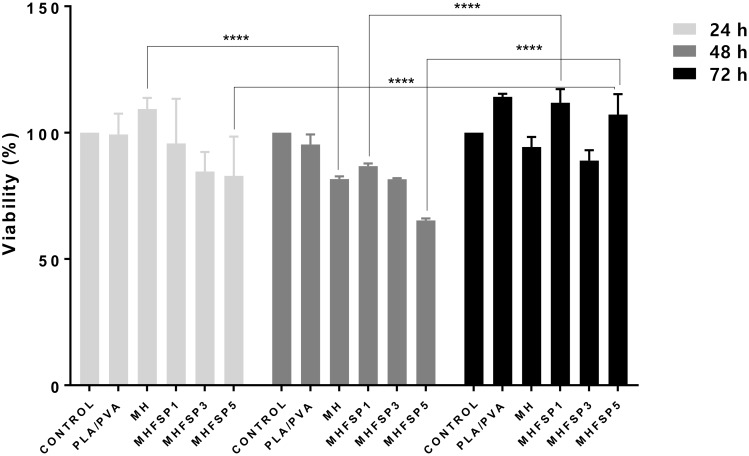
In vitro cytotoxicity assay of PLA/PVA, MH, MHFSP1, MHFSP3 and MHFSP5 samples. Values are the mean standard deviation of three experiments. **** *p* < 0.001 vs. day groups.

As a potential wound dressing material, composite nanofibers should promote cell growth and cell differentiation. The in vitro cytotoxicity tests of coaxial nanofibers toward HaCat cells were evaluated using MTT assays. As shown in Figure 5, the cell viabilities for 72 h were over 80% at all test concentrations, revealing the non-toxicities and good biocompatibilities of composite nanofibers toward cells. Interestingly, we found that neat PVA/PLA nanofibers did not have any significant effect on relative cell viability at 24 h and 48 h time points while in combination with different concentrations of FSP, especially 1% (MHFSP1), it slightly increased the viability, in particularly at 72 h. The cell viabilities of different concentrations had no obvious regularity. After 24 h, the cells may not be fully adherent or have not entered the adaptation process. After 48 h, as the cells enter the adaptation process, some of them may be attached to the material. Some of them may be damaged and dead without attached to the material. Therefore, at the end of 24 h, materials with high viability then may have a low viability after 48 h. However, at the end of 72 h, all test concentrations were found to be above 80% because the cells that were adhering to the material were proliferated. The results suggest that the composite nanofibers did not merely have good biocompatibility but also a promoting effect on cell proliferation after 72 h incubation, indicating that the MH and MHFSP1 composite nanofibers were good biomaterials.

### 3.5. Drug Release Studies and Kinetic Investigation

**Figure 6:**
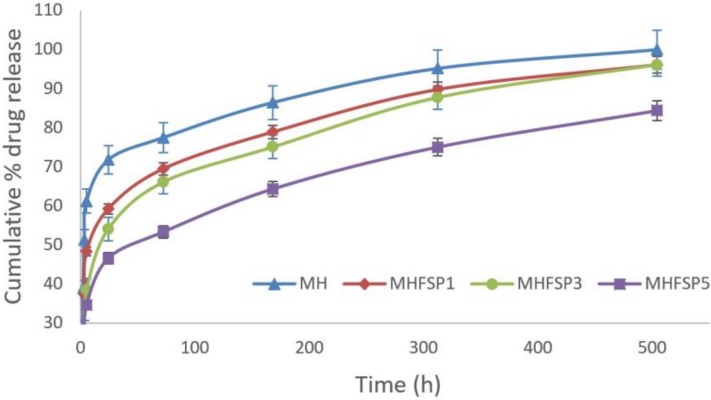
Release profiles of MH, MHFSP1, MHFSP3 and MHFSP5 fibers.

The amount of MH released from the fibers was determined by UV spectroscopy using a pre-determined calibration curve, *y* = 0.1178*x*−0.0083 (*R* = 0.9898) where *y* is the MH concentration (in micrograms per milliliter) and *x* is the solution absorbance at 233 nm which is MH UV absorbance peak. Figure 6 shows the cumulative release rate profiles of MH from coaxial electrospun nanofibers with various FSP concentrations. Experiments of each sample were performed in triplicate, and average values were presented with the error bars, which indicate standard deviation. It is observed from Figure 6 that MH followed a typical dual-stage release profile from the all nanofibers: Firstly, hydrophilic MH showed an initially faster diffusion of coaxial nanofibers (a burst release) and then, a slow trend of sustained release. In fact, it is expected that drug-loaded nanofibers fabricated by coaxial electrospinning generally show sustained release with less initial burst than the blend electrospinning, because the drug is restricted to the core polymer in the core-shell nanofiber while the drug is distributed on the single nanofiber surface. However, faster diffusion or burst release can be observed for the release of hydrophilic small molecule drugs such as MH, as reported previously [35,36]. In the first 24 h, about 75% of the MH was released from the MH nanofibers, while only 59%, 54% and 46% from the MHFSP1, MHFSP3 and MHFSP5 nanofibers, respectively. Furthermore, the nanofibers showed slow and sustained release thereafter and after 21 days where about 99% drug release from MH nanofibers, 96% drug release from MHFSP1 and MHFSP3 nanofibers and 84% drug release from MHFSP3 nanofibers, as shown in Figure 6. These release profiles are in agreement with previous studies of hydrophilic drug release from coaxial nanofibers [37,38]. The results reveal that FSP concentration has a strong effect on initially faster diffusion and sustained drug release. A possible explanation is that the release profile of macromolecular structure of FSP may have formed a suspended site for release of the drug. This improvement can be also attributed to the chemical interactions between MH and FSP content in the nanofiber network. A previous study reported that the increased proportion of the FSP led to increased chemical interactions and thus, a prolonged release behaviour [15]. Clearly as drug carriers, protein loaded nanofiber mats was more stable than protein-free nanofibers.

The release kinetics of MH drug was investigated by fitting with various mathematical models such as zero order, first order, Higuchi and Korsmeyer–Peppas models. The obtained kinetic constants and regression coefficients (*R^2^*) of all samples are given in Table 2. The first order kinetic have better agreement with higher *R^2^* values for all samples than other kinetics. Furthermore, in the Korsmeyer–Peppas model, the value of *n* describes the mechanism of drug release from a polymeric system. For a cylindrical system, 0.45 ≥ *n* corresponds to a Fickian diffusion and 0.45 < *n* < 1 refers the Non-Fickian diffusion [39]. As shown in Table 1, all the *n* values of nanofiber samples are less than 0.45, suggesting MH release from nanofiber samples follow the typical Fickian diffusion mechanism.

### 3.6. Thermal Behaviour

**Figure 7:**
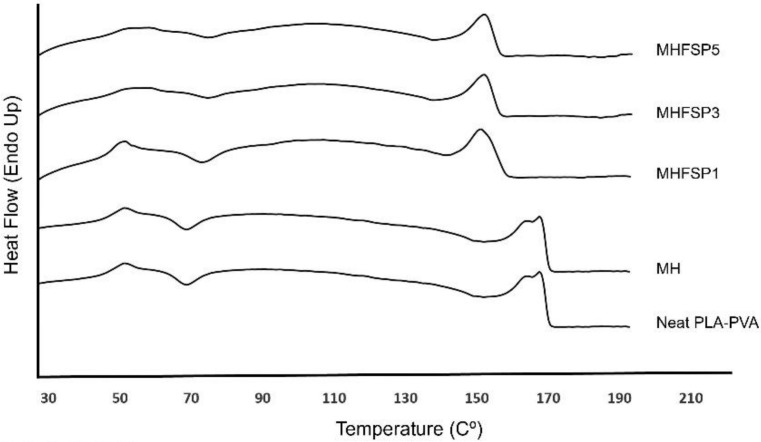
Differential scanning calorimetry (DSC) curves of Neat PLA-PVA, MH, MHFSP1, MHFSP3 and MHFSP5 nanofiber samples.

Differential Scanning Calorimetry (DSC) has been widely used to investigate the crystal structures of electrospun nanofiber mats. It is important to understand the possible structural changes of the biomaterial, such as the change in melting temperature, degree of crystallinity, and enthalpy. The DSC thermograms of the Neat, MH, MHFSP1, MHFSP3 and MHFSP5 coaxial nanofibers are presented in Figure 7. The endothermic peak observed at about 150–165 °C for all the samples was attributed to the crystalline melting (T_m_) of the nanofibers. Neat- and drug-loaded nanofibers showed double peaks at the melting point, the first one at about 163 °C and the second one at 165 °C. According to previous studies, it seems possible that these results are due to the formation of imperfect crystals during the electrospinning process [40]. DSC thermograms of MHFSP1, MHFSP3 and MHFSP5 samples indicate sharp endothermic peaks at about 150 °C. Table 3 shows the melting point and enthalpy of fusion of coaxial nanofibers for comparison. It can be clearly seen that the presence of FSP caused a decrease in melting point, which indicates that it had a reducing effect on the crystallinity of the PLA-PVA coaxial nanofibers. It was also observed that the melting enthalpy of the nanofibers decreased from 23.5 j/g to 10.8 j/g with the addition of FSP. This result supports that the crystallinity of the nanofibers reduced upon the incorporation of FSP proportion. This result is in agreement with a previous study conducted with a different protein [41] and also confirms our previous findings [24].

## 4. Conclusions

In this study, drug and protein loaded nanofibers were successfully fabricated by coaxial electrospinning. Fish Sarcoplasmic Protein (FSP) was produced from Atlantic Bonito and incorported into core polymeric matric at different concentrations. The morphology and chemical compositions of the nanofiber samples were observed using a Scanning Electron Microscopy (SEM) and Fourier Transform Infrared Spectrometer (FTIR). Furthermore, Differential scanning calorimetry (DSC) studies were carried out to investigate the thermal characteristics of the nanofiber mats. According to the results, the coaxial nanofiber mats showed uniform bead-free fiber morphology at an average 650 nm and presented characteristic peaks of the content in the FTIR study. DSC results indicate that the crystallinity of the coaxial nanofibers was reduced with the presence of FSP. The coaxial nanofibers also showed suitable properties for proliferation, and attachment of HaCaT cell lines. Drug release from the coaxial nanofibers were followed by UV spectroscopy and the release profiles were fitted to four popular mathematical equationsm such as zero order, first order, Higuchi and Peppas–Korsemeyer equations to understand the kinetics of drug release. The first order kinetic have better agreement with higher *R^2^* values for all the samples than other kinetics. Indeed, release kinetics of the nanofibers were described on the typical Fickian diffusion mechanism according to the Peppas–Korsemeyer kinetic. Overall, this study reveals that the composite nanofibers had a good potential to be used in the treatment of diabetic ulcers.

## Figures and Tables

**Table 1:** Compositions of the nanofiber samples.

Samples	Core	Shell
MH	5% PVA + MH	8% PLA
MHFSP1	5% PVA + MH + 1% FSP	8% PLA
MHFSP3	5% PVA + MH + 3% FSP	8% PLA
MHFSP5	5% PVA + MH + 5% FSP	8% PLA

MH, metformin hyrochloride; PVA: polyvinyl alcohol; PLA, polylactic acid; FSP, fish sarcoplasmic protein.

**Table 2:** Results of four kinetic models.

Sample	Zero Order		First Order		Higuchi		Korsmeyer-Peppas	
	***R^2^***	***K_o_***	***R^2^***	***K_1_***	***R^2^***	***K_h_***	***R^2^***	***n***
MH	0.6243	0.001	**0.9162**	**−0.0053**	0.79	2.9301	0.7929	0.3327
MHFSP1	0.6535	0.133	**0.9542**	**−0.0025**	0.8407	3.4182	0.7968	0.4764
MHFSP3	0,7339	0.149	**0.9679**	**−0.0025**	0.8944	3.6765	0.8809	0.3911
MHFSP5	0.7775	0.128	**0.9557**	**−0.0016**	0.9191	3.1012	0.9314	0.2751

**Table 3:** Melting temperatures and enthalpy values of nanofibers.

Samples	T_m_ (°C)	ΔHm (j/g)
Neat PLA/PVA	165.5	23.48
MH	165.1	22.96
MHFSP1	150.2	16.3
MHFSP3	150.5	11.1
MHFSP5	150.8	10.8

## References

[1] Xie Z., Paras C.B., Weng H., Punnakitikashem P., Su L.C., Vu K., Tang L., Yang J., Nguyen K.T. (2013). Dual growth factor releasing multi-functional nanofibers for wound healing. Acta Biomater..

[2] Barrientos S., Stojadinovic O., Golinko M.S., Brem H., Tomic-Canic M. (2008). Growth factors and cytokines in wound healing. Wound Repair Regen..

[3] Dahlin R.L., Kasper F.K., Mikos A.G. (2011). Polymeric Nanofibers in Tissue Engineering. Tissue Eng. Part B Rev..

[4] Rieger K.A., Birch N.P., Schi J.D. (2013). Designing electrospun nanofiber mats to promote wound healing—A review. J. Mater. Chem. B.

[5] Kim G., Kim W. (2006). Highly Porous 3D Nanofiber Scaffold Using an Electrospinning Technique. J. Biomed. Mater. Res. Part B Appl. Biomater..

[6] Barnes C.P., Sell S.A., Boland E.D., Simpson D.G., Bowlin G.L. (2007). Nanofiber technology: Designing the next generation of tissue engineering scaffolds. Adv. Drug Deliv. Rev..

[7] Persano L., Camposeo A., Tekmen C., Pisignano D. (2013). Industrial Upscaling of Electrospinning and Applications of Polymer Nanofibers: A Review. Macromol. Mater. Eng..

[8] Zhang Y.Z., Wang X., Feng Y., Li J., Lim C.T., Ramakrishna S. (2006). Coaxial electrospinning of (fluorescein isothiocyanate-conjugated bovine serum albumin)-encapsulated poly(ε-caprolactone) nanofibers for sustained release. Biomacromolecules.

[9] Hu X., Liu S., Zhou G., Huang Y., Xie Z., Jing X. (2014). Electrospinning of polymeric nanofibers for drug delivery applications. J. Control. Release.

[10] Zamani M., Prabhakaran M.P., Thian E.S., Ramakrishna S. (2014). Protein encapsulated core-shell structured particles prepared by coaxial electrospraying: Investigation on material and processing variables. Int. J. Pharm..

[11] Park K.E., Jung S.Y., Lee S.J., Min B.M., Park W.H. (2006). Biomimetic nanofibrous scaffolds: Preparation and characterization of chitin/silk fibroin blend nanofibers. Int. J. Biol. Macromol..

[12] Li M., Mondrinos M.J., Gandhi M.R., Ko F.K., Weiss A.S., Lelkes P.I. (2005). Electrospun protein fibers as matrices for tissue engineering. Biomaterials.

[13] Eltayeb M., Stride E., Edirisinghe M. (2013). Electrosprayed core-shell polymer-lipid nanoparticles for active component delivery. Nanotechnology.

[14] Khadka D.B., Haynie D.T. (2012). Protein- and peptide-based electrospun nanofibers in medical biomaterials. Nanomed. Nanotechnol. Biol. Med..

[15] Stephansen K., Chronakis I.S., Jessen F. (2014). Bioactive electrospun fish sarcoplasmic proteins as a drug delivery system. Colloids Surf. B Biointerfaces.

[16] Verreck G., Chun I., Rosenblatt J., Peeters J., Van Dijck A., Mensch J., Noppe M., Brewster M.E. (2003). Incorporation of drugs in an amorphous state into electrospun nanofibers composed of a water-insoluble, nonbiodegradable polymer. J. Control. Release.

[17] Park J.Y., Lee I.H. (2011). Controlled release of ketoprofen from electrospun porous polylactic acid (PLA) nanofibers. J. Polym. Res..

[18] Huang Z.M., Zhang Y.Z., Kotaki M., Ramakrishna S. (2003). A review on polymer nanofibers by electrospinning and their applications in nanocomposites. Compos. Sci. Technol..

[19] Ferreira M.C., Tuma P., Carvalho V.F., Kamamoto F. (2006). Complex wounds. Clinics.

[20] Lee C.H., Hsieh M.J., Chang S.H., Lin Y.H., Liu S.J., Lin T.Y., Hung K.C., Pang J.H.S., Juang J.H. (2014). Enhancement of diabetic wound repair using biodegradable nanofibrous metformin-eluting membranes: In vitro and in vivo. ACS Appl. Mater. Interfaces.

[21] Chereddy K.K. (2016). PLGA based drug delivery systems: Promising carriers for wound healing activity. Wound Repair Regen..

[22] Ahmed S.M., Ahmed H., Tian C., Tu Q., Guo Y., Wang J. (2016). Whey protein concentrate doped electrospun poly(epsilon-caprolactone) fibers for antibiotic release improvement. Colloids Surf. B Biointerfaces.

[23] Xiao J., Shi C., Zheng H., Shi Z., Jiang D., Li Y., Huang Q. (2016). Kafirin Protein Based Electrospun Fibers with Tunable Mechanical Property, Wettability, and Release Profile. J. Agric. Food Chem..

[24] Sahin Y.M., Su S., Ozbek B., Yücel S., Pinar O., Kazan D., Oktar F.N., Ekren N., Gunduz O. (2018). Production and characterization of electrospun fish sarcoplasmic protein based nanofibers. J. Food Eng..

[25] Hemung B.O., Chin K.B. (2013). Effects of fish sarcoplasmic proteins on the properties of myofibrillar protein gels mediated by microbial transglutaminase. LWT Food Sci. Technol..

[26] Lopez-Enriquez R.L., Ocano-Higuera V.M., Torres-Arreola W., Ezquerra-Brauer J.M., Marquez-Rios E. (2015). Chemical and Functional Characterization of Sarcoplasmic Proteins from Giant Squid (*Dosidicus gigas*) Mantle. J. Chem..

[27] Bazrafshan Z., Stylios G.K. (2019). A novel approach to enhance the spinnability of collagen fibers by graft polymerization. Mater. Sci. Eng. C.

[28] Bazrafshan Z., Stylios G.K. (2018). Custom-built electrostatics and supplementary bonding in the design of reinforced Collagen-g-P(methyl methacrylate-co-ethyl acrylate)/nylon 66 core-shell fibers. J. Mech. Behav. Biomed. Mater..

[29] Colin-Orozco J., Zapata-Torres M., Rodriguez-Gattorno G., Pedroza-Islas R. (2015). Properties of Poly (ethylene oxide)/whey Protein Isolate Nanofibers Prepared by Electrospinning. Food Biophys..

[30] Wongsasulak S., Patapeejumruswong M., Weiss J., Supaphol P., Yoovidhya T. (2010). Electrospinning of food-grade nanofibers from cellulose acetate and egg albumen blends. J. Food Eng..

[31] Sammon C., Mura C., Yarwood J., Everall N., Swart R., Hodge D. (1998). FTIR–ATR Studies of the Structure and Dynamics of Water Molecules in Polymeric Matrixes. A Comparison of PET and PVC. J. Phys. Chem. B.

[32] Suarez-Franco J.L., Vázquez-Vázquez F.C., Pozos-Guillen A., Montesinos J.J., Alvarez-Fregoso O., Alvarez-Perez M.A. (2018). Influence of diameter of fiber membrane scaffolds on the biocompatibility of hPDL mesenchymal stromal cells. Dent. Mater. J..

[33] Chieng B.W., Ibrahim N.A., Yunus W.M.Z.W., Hussein M.Z. (2014). Poly(lactic acid)/poly(ethylene glycol) polymer nanocomposites: Effects of graphene nanoplatelets. Polymers.

[34] Gonçalves R.P., da Silva F.F.F., Picciani P.H.S., Dias M.L. (2015). Morphology and Thermal Properties of Core-Shell PVA/PLA Ultrafine Fibers Produced by Coaxial Electrospinning. Mater. Sci. Appl..

[35] Chou S., Carson D., Woodrow K.A. (2015). Current strategies for sustaining drug release from electrospun nanofibers. J. Control. Release.

[36] Huang X., Brazel C.S. (2001). On the importance and mechanisms of burst release in matrix-controlled drug delivery systems. J. Control. Release.

[37] Doustgani A. (2017). Doxorubicin release from optimized electrospun polylactic acid nanofibers. J. Ind. Text..

[38] Torres-Giner S., Martinez-Abad A., Gimeno-Alcañiz J.V., Ocio M.J., Lagaron J.M. (2012). Controlled delivery of gentamicin antibiotic from bioactive electrospun polylactide-based ultrathin fibers. Adv. Eng. Mater..

[39] Ritger P.L., Peppas N.A. (1987). A simple equation for description of solute release. J. Control. Release.

[40] Guerrini L.M., Branciforti M.C., Canova T., Bretas R.E.S. (2009). Electrospinning and characterization of polyamide 66 nanofibers with different molecular weights. Mater. Res..

[41] He M., Zhang B., Dou Y., Yin G., Cui Y., Chen X., Matsuo M., Huang D., Kim H.-Y. (2017). Fabrication and characterization of electrospun feather keratin/poly(vinyl alcohol) composite nanofibers. RSC Adv..

